# Targeting Lipid—Ion Channel Interactions in Cardiovascular Disease

**DOI:** 10.3389/fcvm.2022.876634

**Published:** 2022-05-06

**Authors:** Emma C. Hudgins, Adam M. Bonar, Thanh Nguyen, Ibra S. Fancher

**Affiliations:** Department of Kinesiology and Applied Physiology, College of Health Sciences, University of Delaware, Newark, DE, United States

**Keywords:** lipids, ion channels, novel therapeutics, dyslipidemia, cardiovascular disease

## Abstract

General lipid-lowering strategies exhibit clinical benefit, however, adverse effects and low adherence of relevant pharmacotherapies warrants the investigation into distinct avenues for preventing dyslipidemia-induced cardiovascular disease. Ion channels play an important role in the maintenance of vascular tone, the impairment of which is a critical precursor to disease progression. Recent evidence suggests that the dysregulation of ion channel function in dyslipidemia is one of many contributors to the advancement of cardiovascular disease thus bringing to light a novel yet putative therapeutic avenue for preventing the progression of disease mechanisms. Increasing evidence suggests that lipid regulation of ion channels often occurs through direct binding of the lipid with the ion channel thereby creating a potential therapeutic target wherein preventing specific lipid-ion channel interactions, perhaps in combination with established lipid lowering therapies, may restore ion channel function and the proper control of vascular tone. Here we first detail specific examples of lipid-ion channel interactions that promote vascular dysfunction and highlight the benefits of preventing such interactions. We next discuss the putative therapeutic avenues, such as peptides, monoclonal antibodies, and aspects of nanomedicine that may be utilized to prevent pathological lipid-ion channel interactions. Finally, we discuss the experimental challenges with identifying lipid-ion channel interactions as well as the likely pitfalls with developing the aforementioned putative strategies.

## Introduction

Ion channels are major regulators of cardiovascular homeostasis. The proper function of vascular cells critically depends on the modulation of ion channels which are involved in the maintenance of vascular tone (1). Cellular lipids are essential to the regulation of ion channel activity, often by direct interaction with ion channels through distinct binding (2, 3). The physiological regulation of ion channels by lipids is necessary to fine-tune ion channel activity, thereby contributing to adjustments in membrane potential (3). In the cardiovascular system, this makes lipids critical regulators of vascular tone, however, it is well-established that elevated blood lipids are associated with an increase in blood pressure, atherosclerosis, and incidence of stroke (4, 5). As such, the dysregulation of lipid levels in the cardiovascular system, as is prominent in cardiovascular diseases of the Western world, represents a major health burden. While the identified cellular mechanisms and pathways affected by lipotoxicity are many (4), the subsequent dysregulation of ion channels repeatedly emerges as a key factor in the perturbation of cardiovascular homeostasis (6–8). Therefore, targeting specific lipid-ion channel interactions has therapeutic potential that, in combination with existing lipid-lowering strategies, may help to curb the progression of severe cardiovascular disease by restoring the appropriate level of channel function and, therefore, cardiovascular homeostasis. Here we will look at several notable examples of lipid-ion channel interactions and discuss which aspects of these interactions are well understood. We will also consider the limitations of current therapeutics and how our current knowledge of the mechanisms of lipid-ion channel interactions could be exploited to develop novel treatments for dyslipidemia. Finally, we discuss experimental difficulties specific to studying lipid-ion channel interactions that may pose a challenge to future research.

### Benefits of Preventing Lipid-Ion Channel Interactions

Generally, a variety of pharmaceutical methods are utilized in dyslipidemic settings in order to lower serum lipid levels, which can prevent advancement of cardiovascular disease. In particular, common drugs include statins, which typically lower levels of LDL-cholesterol, and fibrates, which lower triglyceride levels (9). Others include ezetimibe, which inhibits cholesterol absorption (10), and niacin, which increases HDL-cholesterol and decreases LDL-cholesterol (11). However, non-specific lipid-lowering medications have a variety of clinical drawbacks. Statins in particular are limited to nightly administration due to the nocturnal synthesis of cholesterol, and have negative side effects such as myopathy, hepatotoxicity, and medication resistance (12). Bile-acid binding resins increase hepatic cholesterol uptake thereby decreasing cholesterol levels in the blood (13) but are associated with gastrointestinal side effects due low absorption in the intestines, leading to fecal excretion and high dosage frequency/concentration (14). Therefore, development of treatments targeting the specific mechanisms leading to vascular dysfunction is warranted. For a brief summary of existing therapeutics, see Table 1.

**Table 1:** Traditional vs. putative methods of targeting lipid-ion channel interactions.

		**Advantages**	**Disadvantages**	**References**
**Traditional Methods**	Statins	- Clinically effective at reducing risk of cardiovascular disease through serum LDL reduction	- Limited to night-time administration - Medication resistance - Side effects- Non-specific	(9, 12)
	Bile-Acid Binding Resins	- Clinically effective at reducing risk of cardiovascular disease through serum cholesterol reduction - Minimal and limited systemic side effects	- Gastrointestinal side effects	(13, 14)
**Putative Methods**	Peptides	- Highly specific	- Short half life - Immunogenic - Low oral bioavailability - High risk for side effects	(24, 25)
	Monoclonal Antibodies	- Highly specific - Consistent pharmacokinetics - Limited effect on nervous system - Long half-life	- Expensive - Difficult to produce - May not effectively bind to ion channels due to structural impediments	(24, 26)
	Nanobodies	- Smaller size allows for greater access to hard-to-reach epitopes - Intracellular generation - Can target cytosolic and extracellular epitopes	- High renal clearance leads to low retention - Potential for nephrotoxicity	(24, 27)
	Nanomaterials	- Wide range of functionality - Long half-life	- Possible unwanted accumulation - Potentially hazardous - Possibly limited to use in inflammatory conditions	(28, 29)

While the regulation of ion channel function by lipids is well established (3), a few have been shown to demonstrate particular relevance to vascular dysfunction (7, 8). However, the associations between lipids and ion channels are complex and may involve the direct binding of lipids to ion channels and/or non-specific changes to the plasma membrane (2, 15). These two mechanisms are not mutually exclusive and may both contribute to modulation of ion channel function. Furthermore, the role of additional proteins (e.g., ancillary ion channel subunits) in the plasma membrane may alter the functional effect of lipids (16). However, the apparent specificity of many lipid-ion channel interactions may permit the development of therapeutics that specifically bind to and prevent these specific interactions, thereby preventing the binding of inhibitory lipids to channel proteins. Ideally, this therapeutic would not influence ion channel function but rather allow ion channel activity to proceed in the presence of what would otherwise be an inhibitory concentration of lipids.

**Figure 1:**
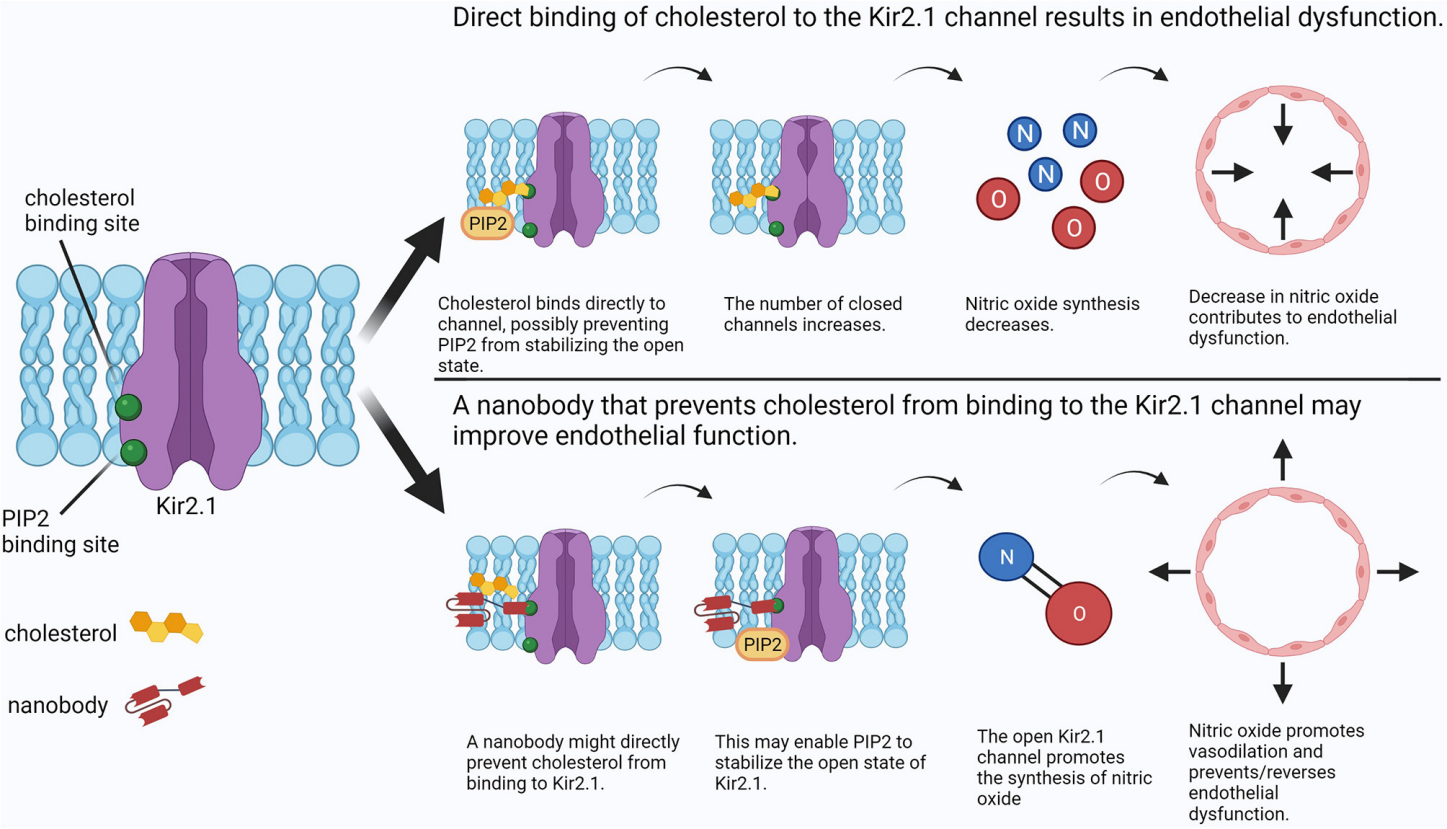
Comparison between cholesterol-induced Kir2 channel inhibition and a putative nanobody therapeutic preventing cholesterol-Kir2 interaction. In the presence of elevated cholesterol, cholesterol may bind directly to Kir2, preventing PIP2 from stabilizing the open state and resulting in channel inhibition. Consequently, nitric oxide synthesis is decreased resulting in endothelial dysfunction. A possible treatment may use an antibody to directly prevent cholesterol from binding to Kir2 while still allowing channel regulation by PIP2. Physiological synthesis of nitric oxide is restored, preventing or reversing endothelial dysfunction. Created with BioRender.com.

One such lipid-ion channel interaction of interest is that of cholesterol with Kir2, an inwardly-rectifying potassium channel expressed in numerous tissues (17). This channel appears to be involved in mechanosensing in endothelial cells and plays an important role in the regulation of vascular tone by promoting nitric oxide synthesis (7, 18). Recent studies showed that an increase in cellular cholesterol has an inhibitory effect on Kir2 channels by decreasing Kir2 channel function rather than expression. Interestingly, a reduction of flow-induced vasodilation in a high-cholesterol environment was completely reversed by both the depletion of cholesterol and the overexpression of Kir2 in mouse primary mesenteric artery endothelial cells (7), suggesting that both preventing cholesterol binding and promoting Kir2 function may be effective approaches to preventing and treating endothelial dysfunction in the presence of hypercholesterolemia. Several studies compared the effects of cholesterol with its isomers and concluded that cholesterol likely interacts directly with the channel protein to cause inhibition rather than altering the physical properties of the lipid bilayer and affecting the channel indirectly (19, 20). This process may involve phosphatidylinositol 4,5-bisphosphate (PIP2) (21, 22), the presence of which stabilizes the open state of the channel (23). It was suggested that cholesterol weakens the interaction of PIP2 with Kir2 channels, resulting in instability of the open state (22). As cholesterol binds to a distinct site from that of PIP2, it was proposed that cholesterol-Kir2 interactions caused a structural change in Kir2 that opposes that of PIP2 binding, resulting in the inability of PIP2 to stabilize the open state (Figure 1, top panel) (21). Therefore, targeting cholesterol-Kir2 interactions to prevent cholesterol from interacting with Kir2 channels would require generating a small molecule or drug that allows or promotes PIP2-Kir2 channel interactions (Figure 1, bottom panel).

The interaction of large-conductance, calcium-activated potassium (BK) channels with cholesterol has also received considerable attention. BK channels are major regulators of smooth muscle contraction in multiple tissue types in the colon (30), the urinary bladder (31) and notably the arteries, where activation of the channel results in vasodilation (32). In arterial smooth muscle cells, the pore of the channel is composed of alpha pore-forming subunits and beta auxiliary subunits which increase sensitivity to Ca^2+^. A recent study showed that cholesterol has a direct inhibitory effect on the BK channel when only the alpha subunits are present. In contrast, elevated cholesterol activates BK channels when beta subunits are also expressed. The authors concluded that the cholesterol-mediated activation appears to be induced by increased trafficking of the beta subunit to the plasma membrane which results in increased sensitivity of BK to Ca^2+^ (16). The alpha subunit contains several conserved cholesterol recognition amino acid consensus (CRAC) motifs that may constitute the binding sites by which cholesterol inhibits BK channel activity in the absence of beta subunits, while the beta subunits also contain two CRAC motifs (32). Although the role that CRAC motifs play in cholesterol binding and effects on channel function in general is debated (19, 32, 33), the increase in cholesterol-mediated beta subunit trafficking is likely driven by a separate cellular pathway that is triggered by elevated cholesterol. This putative mechanism, which potentially serves as a protective negative feedback mechanism in the presence of high cholesterol, illustrates the potential benefit of novel therapeutics that target specific lipid-channel subunit binding. For example, in contrast to general lipid lowering therapies, a treatment specifically preventing cholesterol interaction with the BK alpha subunit at the binding site could be an effective method for preventing undesired consequences of dyslipidemia while permitting cholesterol-mediated beta subunit trafficking, thus promoting BK activation and vasodilation.

While ion channels are not currently a traditional target in dyslipidemia, the dysfunction of ion channels is implicated in the development of severe cardiovascular disease in dyslipidemic settings (7, 8). The well-established effects of lipids directly inhibiting specific vascular ion channels warrants investigation into preventing these specific lipid-ion channel interactions to prevent the progression of disease. Therefore, novel approaches in targeting lipid-ion channel interactions represent potential ways to restore proper ion channel function in dyslipidemic populations. For a detailed review of the interaction of cholesterol with Kir2 and BK channels as well additional ion channels not discussed here, including the nicotinic acetylcholine receptor (nAChR) channel and the transient receptor potential (TRP) channels, we refer the reader to (19, 34–36).

### Putative Methods to Target Lipid-Ion Channel Interactions

Traditional pharmaceutical methods that target lowering blood lipid levels are clinically effective in treating cardiovascular disease (37). However, the drawbacks to lipid lowering therapies discussed above limit the overall efficacy and adherence to these drugs (38). While ion channels have not been widely studied as targets in dyslipidemia, an increasing body of evidence suggests that preventing lipid-ion channel interactions that promote inhibition of the channel may serve to restore ion channel-mediated control of vascular tone, limiting or preventing the progression of disease. In the following sections we discuss possible avenues that may be suitable for targeting specific lipid-ion channel interactions and discuss potential drawbacks to these presently putative methods.

#### Peptides

The ability for researchers to modify peptides to harness desirable effects presents a putative approach in preventing lipid-ion channel interactions and experimental evidence supports this avenue. A recent study showed that blocking lipid-TRPV1 (transient receptor potential vanilloid 1) channel interactions with a peptide prevented diabetes-induced endothelial dysfunction in mice (39). In this instance, lipid-mediated channel activation promoted pathophysiology and a peptide specifically targeting the lipid-channel interaction at the TRP box site prevented endothelial dysfunction, indicating that the presence of other conditions (e.g., diabetes) may influence which lipid-ion channel interactions should be specifically targeted in the presence of dyslipidemia. For instance, in the absence of diabetes, endothelial TRPV1 activation may promote nitric oxide production and contribute to vasodilation (40). Both cholesterol and oleic acid inhibit the channel through direct interactions, though membrane effects have not been ruled out (41). The tarantula double knot toxin (DkTx) targets TRPV1, activating the channel at low doses (39). Cholesterol has been shown to bind in the S5 helix of TRPV1, and DkTx to the S6 and S5 helices (42, 43), suggesting that DkTx could be used to simultaneously promote channel activation while preventing cholesterol-induced suppression of TRPV1 channels by blocking cholesterol binding at the S5 site. Venom peptides, well known for having specific effects on a variety of ion channels at low concentrations, have been utilized for a variety of medical advances such as the production of the angiotensin-converting enzyme inhibitor that currently treats hypertension (38). Produced by venomous animals, these peptides are potent and specific, allowing for many potential functions, including the activation/inhibition of ion channels (24). However, venom peptides have a short half-life which would entail a higher dosing frequency, complicating the noxious effects on tissues. Moreover, these peptides have the potential to be very immunogenic, leading to potential adverse reactions. Lastly, venom peptides do not have much oral bioavailability, which can cause complications in drug administration (25). Modifications made to venom peptides such that selective binding to ion channels can occur without destroying tissue is one example of how peptides should be modified to optimize desired benefits while mitigating harmful effects. Future research aimed at reducing toxicity and immunogenicity, increasing oral bioavailability, and targeting specific ion channel moieties through manipulating peptide length/sequence may allow for effective clinical usage of venom peptides to target lipid-ion channel interactions.

#### Monoclonal Antibodies

Monoclonal antibodies (mAbs) have been widely exploited to treat a variety of conditions, from cancer to COVID-19 (44, 45), and their use in manipulation of ion channel function has grown considerably in recent years (26). mAbs also have the potential to be utilized in targeting lipid-ion channel interactions, such as through specific binding of epitope(s) on the ion channel (26). Moreover, mAbs generated to directly target elevated lipids could potentially be used to bind a critical concentration of a specific lipid that would prevent interaction with the ion channel yet allow a physiological concentration of lipids to exist. Many small molecules/drugs are also disadvantaged in that they may have a short half-life. In contrast, mAbs have a relatively extended half-life ranging from days to weeks, which could lead to lower dosing frequencies (24). Additionally, mAbs are highly specific, which decreases the likelihood of off-target binding, and have little variability in patient pharmacokinetics which allows for consistent dosing administrations (26). However, mAbs are expensive and difficult to generate. Furthermore, small target epitopes may lack the proper immunogenicity for mAbs, or if the target has bulky structures present, these structures may physically impede the binding of mAbs (26). Additionally, producing mAbs that do not impede channel function may be impractical. Generating mAbs that recognize a lipid antigen may be the most useful way in using mAbs to prevent specific lipid-ion channel interactions. Thus, these challenges presented by mAbs may be best addressed by nanobodies.

#### Nanomedicine

##### Nanobodies

Nanomedicine represents a groundbreaking frontier for the future of healthcare. From enhanced drug delivery to early cancer detection, the possibilities are vast (46). In this regard, nanobodies (Nbs) have immense therapeutic potential in targeting lipid-ion channel interactions in individuals afflicted by hyperlipidemia. Nbs are small (15 kDa; 2–3 nm in diameter) immunoglobulins with similar functions to mAbs (47). As described above, a common issue with mAbs is their large size, which can reduce access to epitopes. Nbs, on the other hand, are able to access hard-to-reach protein sequences along with being able to passively diffuse through membranes, allowing for access to membrane, cytosolic, and extracellular epitopes (24). An increased range of sequences can be targeted by Nbs, which allows for greater possibilities of impacting lipid-ion channel interactions. For example, and as shown in Figure 1 (bottom panel), Nbs could be generated to bind to a cholesterol-binding region on an ion channel, acting as a competitive inhibitor of cholesterol, while allowing the ion channel to function. In contrast to mAbs, Nbs only require one domain for keeping target specificity. This allows Nbs to be functional in more environments such as the cytoplasm and membrane, where fatty acids have been shown to inhibit specific ion channels. For instance, it has been shown that cytoplasmic accumulation of long chain coenzyme A, a fatty acid metabolism intermediate, leads to the inhibition of Kir channels by direct binding (48–50). In this case, Nbs could hypothetically target the cytoplasmic binding site of these intermediates. However, more research is needed to determine if targeting such epitopes of ion channels with Nbs will show high efficacy, or if inducing genetic expression of Nbs that target ion channels is a possible avenue (24). Additionally, Nbs have a fast renal clearance which can ultimately lead to kidney toxicity (27). Nevertheless, Nbs provide a wide array of opportunities to target lipid-ion channel interactions that could have a potential role in ameliorating dyslipidemia-induced cardiovascular disease.

##### Nanomaterials

Along with nanobodies, the use of nanomaterials (Nms) represents another approach in the evolving field of nanomedicine. Nms are classified as materials or chemical substances that exist on a nanoscale of 1–100 nanometers (51). Nms have a wide array of functions and many unique structures along with an extended half-life to allow for reduced dosing regimens. Nms have been used to block ion channels by using hydrophobic interactions and molecular complementarity, causing ion flow to be reduced or ceased (28). Manipulating Nms to prevent lipid-ion channel interactions without impacting channel function would be a necessary alteration. Thus, Nms would need to be engineered to target binding sites of ion channels, acting as competitive inhibitors. While Nms have already been shown to bind and interact with ion channels and are therefore a potential approach in preventing lipid-ion channel interactions, Nms can also present a variety of clinical challenges. Dosing can become an issue, as it remains to be determined how to prevent Nm accumulation at non-targeted sites. Additionally, potential risks of Nms have been studied, such as the potential for induction of pathological states like pneumoconiosis and neurological disorders (28, 29). Future research aimed at abrogating the health risks associated with Nms is necessary prior to considering the use of Nms as a therapeutic use in targeting lipid-ion channel interactions in dyslipidemic populations.

For a brief overview of possible treatment strategies, see Table 1.

## Discussion

### Identifying Lipid-Ion Channel Interactions, Experimental Challenges, and Theoretical Approaches

The generation of novel therapeutics such as those that may benefit ion channel function in dyslipidemia first requires a diligent and thorough investigation of the (patho)physiological consequences of the lipid-ion channel interaction in question. The nature of lipid-ion channel interactions poses unique challenges that can create roadblocks for the development of cardiovascular treatments. Furthermore, is it important to note that preventing lipid-ion channel interactions may produce unwanted effects that interact with other areas of lipid function and regulation that are important for normal biologic processes, especially with a non-specific therapeutic. For example, Kir2 channels prevent spontaneous electrical activity in excitable cells, such as cardiomyocytes (52). Inhibition of cholesterol binding in Kir2 channels could pose an issue if specific tissues cannot be targeted. In this case, cardiac Kir2 channels could be impacted and lead to arrhythmias (35). Tissue- or cell-specific targeting is just one of a variety of potential challenges that these putative methods will need to address.

While computational modeling has contributed much to the identification of potential lipid-ion channel binding sites (2, 23, 53), fundamental limitations exist in the reduction of the cellular environment to simple membranes and poor sampling (53). Additionally, the binding of a lipid to an ion channel protein does not necessarily cause a predictable functional result. Though advances in dynamic simulations, wherein protein conformation changes in the presence of lipid contact can be observed, are rapidly progressing (54), most of the work to date was conducted on static protein structures, which does not represent the dynamic conformation changes of ion channels *in vivo*. Despite these pitfalls, computational modeling serves as an excellent, albeit time-consuming, foundational approach to identifying large numbers of lipid-ion channel interaction sites.

Following the identification of potential lipid-ion channel interaction sites by computational modeling, these sites are further explored *in vitro*, most commonly by a mutagenesis screen. In the case of investigating a specific ion channel, multiple mutations to a variety of putative lipid binding sites can be simultaneously and rapidly screened using an automated patch clamp system to ensure that a given mutation (i) does not drastically influence channel function and (ii) is functional in the presence of the otherwise inhibitory lipid of interest. However, the automated patch systems used for screening large numbers of cells at once are expensive and often researchers are left to systematically test each mutation individually in patch clamp experiments. This, along with generating the mutant plasmid constructs prior to electrophysiological testing, represents another time-consuming approach that often results in the identification of only a few mutations that confer the desired effects.

Introducing mutations with the desired effects into an animal model is the gold standard to identify if rendering an ion channel insensitive to a specific lipid promotes a physiological rescue effect on vascular function in the presence of dyslipidemia. The arrival of CRISPR technologies has drastically reduced the time and cost to generate transgenic mice, however, to investigate tissue-specific effects use of the Cre-loxP method is, at this time, required and is much more costly and time consuming. While it may seem time/cost efficient to directly screen for peptides and/or nanomaterials that may prevent lipid binding in lieu of mutagenesis studies, ensuring that preventing the specific lipid-ion channel interaction by first confirming that a physiological consequence arises from rendering the channel insensitive to lipid-induced inhibition is a necessary step in identifying novel lipid-ion channel interactions that have pathological consequence.

Generating therapeutics that prevent specific lipid-ion channel interactions with minimal adverse events presents numerous challenges, many of which are unique to the molecule of choice, with each therapeutic avenue considered requiring stringent validation for efficacy and safety. Several iterations of a theoretical therapeutic (e.g., manipulated sequences of a venom peptide predicted to bind the desired ion channel lipid binding site) may proceed in a similar fashion beginning with computational modeling to show that the molecule binds to the desired ion channel sequence and prevents lipid binding/interaction with the channel. *In vitro* screening of successful versions of the therapeutic identified in the modeling may follow to identify which versions of the therapeutic molecule restores ion channel function in the presence of the inhibitory lipids. Work in animal models will then need to determine (i) whether the therapeutic promotes a restoration of ion channel and vascular function *ex vivo/in vivo*, (ii) dosing required for maximum physiological benefit without adverse events *in vivo*, (iii) routes of administration, and (iv) clearance/toxicity to off target tissues. Ultimately, clinical trials will determine the validity of such a theoretical approach in preventing lipid-ion channel interactions to promote vascular health, perhaps in combination with lipid lowering therapies, and take the field into directions whereby differences in distinct patient populations (e.g., stratified by race, ethnicity, sex, age, and metabolic/obesity profile) can be targeted and treated. Finally, the identification of ion channel mutations in patient populations that may affect lipid binding and channel function should also be considered as the field progresses.

## Data Availability Statement

The original contributions presented in the study are included in the article/supplementary material, further inquiries can be directed to the corresponding author/s.

## Author Contributions

EH, AB, and IF conceptualized and wrote the manuscript with editing by EH, AB, TN, and IF. EH and AB generated the figure and table which were edited by TN and IF. All references were checked for accuracy by TN and IF. All authors contributed to the article and approved the submitted version.

## Funding

This work was supported by the National Institutes of Health (NIH) Grant P20GM113125-6564 (IF), the Delaware IDeA Network of Biomedical Research Excellence program, with NIH Grant P20GM103446 from the NIH and the State of Delaware (IF), and a University of Delaware General University Research grant (IF).

## Conflict of Interest

The authors declare that the research was conducted in the absence of any commercial or financial relationships that could be construed as a potential conflict of interest.

## Publisher's Note

All claims expressed in this article are solely those of the authors and do not necessarily represent those of their affiliated organizations, or those of the publisher, the editors and the reviewers. Any product that may be evaluated in this article, or claim that may be made by its manufacturer, is not guaranteed or endorsed by the publisher.
